# The impact of the Onco*type* DX Recurrence Score on treatment decisions and clinical outcomes in patients with early breast cancer: the Maccabi Healthcare Services experience with a unified testing policy

**DOI:** 10.3332/ecancer.2013.380

**Published:** 2013-12-17

**Authors:** Nava Siegelmann-Danieli, Barbara Silverman, Aviad Zick, Anat Beit-Or, Itzhak Katzir, Avi Porath

**Affiliations:** 1 Maccabi Healthcare Services, 27 Hamered Street, Tel Aviv 68125, Israel; 2 Hadassah Medical Center, Ein Kerem, Jerusalem 91120, Israel

**Keywords:** biomarker, breast cancer, chemotherapy, health policy, personalised medicine

## Abstract

The Onco*type *DX Recurrence Score is a validated prognosticator in oestrogen receptor positive (ER+) breast cancer. Our retrospective analysis of a prospectively defined cohort summarises the clinical implications associated with Onco*type *DX testing according to the Maccabi Healthcare Services (MHS) policy. The MHS eligibility criteria for testing included ER+ N0/pN1mic invasive tumours, discussion of test implications with an oncologist, ductal carcinoma 0.6–1 cm Grade 2–3, HER2 negative ductal carcinomas with 1.1–4.0 cm Grade 1–2, or lobular carcinoma. Large (> 1 cm) Grade 3 tumours could have grade reassessed. We linked Recurrence Score results with patients’ information and used chi-squared tests to assess the associations thereof. Between January 2008 and December 2011, tests were performed on 751 patients (MHS-eligible, 713); 54%, 38%, and 8% of patients had low, intermediate, and high Recurrence Score results, respectively. Recurrence Score distribution varied significantly with age (*P *= 0.002), with increasing Recurrence Score values with decreasing age. The proportion of patients with high Recurrence Score results varied by grade/size combination and histology, occurring in 32% of small (≤ 1 cm) Grade 3 and 3% of larger (1.1–4 cm) Grade 1 ductal tumours and only in 2% of lobular carcinomas. Chemotherapy was administered to 1%, 13%, and 61% of patients with low, intermediate, and high Recurrence Score results, respectively (*P *< 0.0001), but only to 2% of intermediate score patients ≥ 65 years. Luteinising-hormone-releasing hormone agonists with tamoxifen were used in 27% of low Recurrence Score patients ≤ 50 years. With a median follow-up of 26 months, no systemic recurrences were documented, whereas four patients exhibited locoregional recurrences. In summary, in this low-to-moderate risk patient population, testing identified 46% of patients as intermediate/high risk. Treatment decisions were influenced by Recurrence Score results and patients’ age. The current MHS policy seems to achieve the goal of promoting chemotherapy use according to the test results in a prespecified patient population.

## Background

Molecular testing in oncology is a highly evolving field with a potential impact on early detection, diagnosis, and risk assessment, as well as on establishing prognosis and predicting both responses to therapy and drug toxicity. Tests that are necessary for treatment decisions, such as HER2 levels in breast cancer (for HER2-directed therapies) and anaplastic lymphoma kinase rearrangement in lung adenocarcinoma (for crizotinib therapy), are scored high for clinical utilities and are covered by most modern insurance plans. However, other molecular tests that are intended to assist in the prognostic and/or predictive evaluation of early breast tumours, such as the Onco*type *DX breast cancer assay (Genomic Health Inc., Redwood City, California, United States) and MammaPrint 70 gene assay (Agendia, Amsterdam, The Netherlands), may be considered to have lower scores for clinical utility and, therefore, are not uniformly covered by providers.

Maccabi Healthcare Services (MHS) is the second largest health maintenance organisation (HMO) in Israel, insuring close to two million members and caring for over 8,700 new cases of adults with nonhaematology invasive tumours annually. Among these cases, approximately 1,000 are breast cancer patients, the majority of which (97%) are diagnosed with nonmetastatic disease (MHS members participate in the National Breast Cancer Early Detection Programme in Israel). HMO resources in Israel have been increasingly strained since 1995, with changes in state reimbursement following the introduction of the Israeli National Health Insurance Law. While modern oncology drugs are often covered under the law, certain molecular technologies, including breast cancer profiling, are not reimbursed by the state. In January 2008, MHS elected to approve and completely cover molecular profiling for newly diagnosed breast cancer patients according to a prespecified policy. This policy aimed to identify patients whose likelihood (prior to molecular profiling) of being offered chemotherapy in addition to hormonal therapy is low-to-moderate and whose lives may be saved by early chemotherapy use. Onco*type *DX, a 21-gene real-time reverse transcription polymerase chain reaction assay, that provides numerical results in the form of a Recurrence Score [1], was elected as the molecular test of choice, as it can be performed on fixed paraffin-embedded tissues, is considered highly accurate with repetitive tests [2, 3], is supported by an extensive database [4], and its prognostic and predictive utility in oestrogen receptor positive (ER+) node negative patients has been demonstrated in several large cohorts using prospectively designed, retrospective analyses of archival samples, with prespecified statistical analysis plans (NSABP B-14 and NSABP B-20 [1, 5]; there are now more validation studies with consistent results) [6–10].

This article summarises the experience of MHS with Onco*type *DX testing from 2008 to 2011, and addresses the association of the Recurrence Score results with clinical and pathological features, treatment approaches, and clinical outcomes.

## Methods

### Patients

This retrospective analysis of a prospectively defined cohort evaluated all MHS patients who underwent Onco*type *DX testing from 1 January 2008 to 31 December 2011. All tests were approved by MHS headquarters. The approval policy required patients to meet the following criteria: (a) diagnosis of ER+ primary breast cancer and node negative or microscopic nodal involvement only; (b) having discussed the implications of the test with an oncologist and being prepared to undergo chemotherapy should it be recommended; and (c) having ductal carcinoma 0.6–1.0 cm with Grade 2–3 histology (if HER2 positive, not being considered for immediate trastuzumabcontaining therapy), OR HER2 negative 1.1–4.0 cm with Grade 1–2 ductal histology, OR infiltrating lobular tumours. Large (> 1.0 cm) ductal tumours of Grade 3 histology were not approved; however, the grade could be reassessed and the test was approved if histology grade was then determined as 1–2. Genetic predisposition to breast cancer was not taken into consideration for testing approval (MHS headquarters personnel had no information on patients’ BRCA 1/2 mutation status at the time of testing approval). In rare cases, testing was also approved for patients not fulfilling the MHS criteria, mostly due to unique medical conditions. The study was approved by the Ethics Committee of Assuta Hospital. All patients signed informed consent forms prior to Onco*type* DX testing.

### Data collection

Individual information for all breast cancer patients who underwent Onco*type *DX testing was extracted from the MHS database, including pathology information, which was reviewed by an oncology physician, and demographic, vital status, pharmacy purchasing, and hospital claims data, which were all extracted electronically for the period from the date of testing through 30 June 2012. Ki-67 data were available for patients who had a core biopsy or breast surgery at MHS-affiliated facilities (i.e., when tumour samples were sent to the MHS central pathology lab for analysis). There, Ki-67 levels were measured using an anti-Ki-67 antibody (SP6, Thermo Scientific, Rockford, Illinois, United States) and the BenchMark XT staining platform (Ventana, Tucson, Arizona, United States). Teva Pharmaceutical Industries Ltd, the distributer of Onco*type *DX testing in Israel, provided the Recurrence Score results, which were categorised as follows: low (< 18); intermediate (18–30); and high (≥ 31). The patients may have been treated by any oncologist in Israel (either affiliated with MHS or not) according to their personal preferences. The treatments received (chemotherapy, endocrine therapy, or both) were ascertained by combining data from pharmacy purchases and hospital claims. The current analysis defined patients as having received systemic chemotherapy if chemotherapy-specific hospital bills were submitted for services provided within 12 months of testing. Pharmacy purchasing records provided information on hormonal therapy with tamoxifen; aromatase inhibitors (AIs), including anastrozole, letrozole, and exemestane; and luteinising-hormonereleasing hormone (LHRH) agonists. The clinical documents for all patients who died or had subsequent (≥ 12 months) chemotherapy or late mastectomy (over six months from the date of testing) were reviewed to assess systemic and locoregional recurrences.

### Analyses

The entire cohort was used to evaluate the relationship between Recurrence Score categories and treatments received. The relationship between score categories and clinicopathological characteristics was limited to the 95% of patients who met the MHS eligibility criteria for testing. The Mantel–Haenszel chi-squared test was used to test for the association between score and age categories and the Cochran– Mantel–Haenszel row mean score chi-squared test was used to test for the association between score category and nominal clinicopathological characteristics and treatments received. Where appropriate, *post hoc *pairwise comparisons were corrected for multiple testing by the Bonferroni method. A multivariable logistic regression model was used to assess the probability of receiving chemotherapy as a function of score category, age, and the interactions thereof. All analyses were conducted using SAS statistical software version 9. 2 (SAS Institute Inc., Cary, North Carolina, United States); *P* < 0.05 was considered significant.

## Results

### Patient population

A total of 751 MHS patients who underwent Onco*type *DX testing between 1 January 2008 and 31 December 2011 were included, 713 of whom (95%) met the MHS criteria for testing. The majority of our cohort (485 patients, 64.6%) was 45–64 years of age, 138 patients (18.4%) were 18–44 years of age, and 128 patients (17.0%) were ≥ 65 years. Recurrence Score distribution showed that 407 patients (54.2%) had a low Recurrence Score result, 285 patients (37.9%) had an intermediate Recurrence Score result, and 59 patients (7.9%) had a high Recurrence Score result.

Recurrence Score distribution remained stable over the years (*P *= 0.27; chi-squared test, data not shown). It varied significantly, however, with age (*P* = 0.002; Mantel–Haenszel chi-squared test), with increasing Recurrence Score values with decreasing age (Table 1).

### Recurrence score distribution and pathological characteristics

Analysis was conducted for the MHS-eligible patients. The majority (590 patients, 82.7%) had invasive ductal carcinoma (IDC) not specified, 21 patients (3.0%) had favourable IDC histological subtypes (mucinous, tubular, and medullary [11]), and 102 patients (14.3%) had invasive lobular carcinoma (ILC) alone or mixed with IDC (87 patients with ILC alone, 15 with mixed; all considered ILC for the current analysis). The Recurrence Score distribution differed significantly between the histological subtypes (*P *= 0.02; Cochran–Mantel–Haenszel row mean score test; Table 1). In pairwise comparisons, the only significant difference was between the IDC not specified and the ILC groups (*P *= 0.017; Cochran–Mantel–Haenszel row mean score test using the Bonferroni correction). Intermediate and high Recurrence Score rates in the IDC not specified group were 38.6% and 8.1%, respectively, and in the ILC group, these rates were 32.4% and 2.0%. The high Recurrence Score category in the ILC group referred to only two patients, both of whom had microscopic nodal involvement and primary tumour size ≥ 2 cm. Of note, Recurrence Score distribution in the IDC favourable group was not significantly different from that in the IDC not specified group (*P* = 0.86).

When only IDC patients were assessed, grade/size combination was significantly associated with Recurrence Score results: despite performing the test only for small (≤ 1 cm) Grade 3 tumours, high Recurrence Score results occurred in 32.1% of those cases, as opposed to 3.0–8.8% of Grade 1–2 tumours sized 0.6–4.0 cm (*P *< 0.0005 for Grade 3 and ≤ 1 cm versus all other grade/size combinations; Cochran–Mantel–Haenszel row mean score test using the Bonferroni correction; Table 1).

Next, we examined the association between the Recurrence Score results and the level of the proliferation biomarker Ki-67, which was available for 389 patients (55.0%). There was a weak but statistically significant positive correlation between the two parameters (*r *= 0.32, 95% confidence interval, 0.23–0.41; Pearson correlation). The Recurrence Score distribution differed significantly between patients with Ki-67 ≤ 15% and > 15% (*P *< 0.0001; Cochran–Mantel–Haenszel row mean score test; Table 1), but there was a wide distribution of Recurrence Score results in both groups.

### Recurrence score results and treatments received

**Figure 1.**
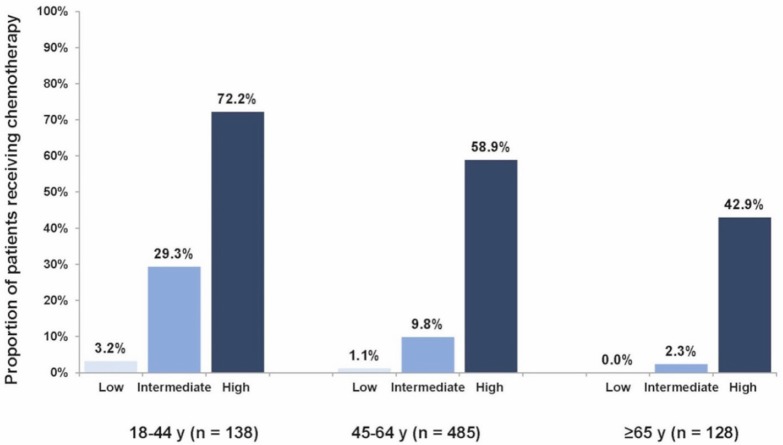
Proportions of patients receiving chemotherapy by age group and Recurrence Score category. Increase from each Recurrence Score level to the next (P < 0.0001); decrease from 18–44 years to 45–64 years (P = 0.0043), from 45–64 years to ≥ 65 years (P = 0.039); logistic regression.

Of the 751 patients in our cohort, 18 patients (2%) received no chemotherapy and did not purchase any hormonal agents. Altogether, 77 patients (10%) received systemic chemotherapy. High Recurrence Score patients received chemotherapy at a greater rate than intermediate and low Recurrence Score patients (61%, 13%, and 1%, respectively; *P *< 0.0001; chi-squared test) overall and even in the older age group (≥ 65 years), in which 43% of high Recurrence Score patients received chemotherapy. For intermediate Recurrence Score patients, older age was significantly associated with a decreased use of chemotherapy (29%, 10%, and 2% of patients 18–44, 45–64, and ≥ 65 years received chemotherapy, respectively; *P* < 0.0001 for comparing chemotherapy use across age groups; chi-squared test; Figure 1).

In a multivariable logistic regression analysis in which the probability of receiving chemotherapy was modelled as a function of Recurrence Score category, age, and the interaction thereof, the interactions were found to be nonsignificant. Recurrence Score category and age group were found to be independent predictors of chemotherapy use (Table 2).

Of the 751 patients in our cohort, 730 patients (97%) purchased endocrine therapy agents (tamoxifen, AIs, LHRH agonists, or their combinations) from MHS-owned/affiliated pharmacies. Overall, tamoxifen was purchased for 659 patients (88%), AIs for 238 patients (32%), and LHRH agonists for 90 patients (12%). For the most part, the purchase/use of endocrine therapy was similar across the Recurrence Score categories; the range of endocrine therapy use across the Recurrence Score categories was 78–90% for tamoxifen, 31–34% for AIs, and 9–13% for LHRH agonists.

**Figure 2.**
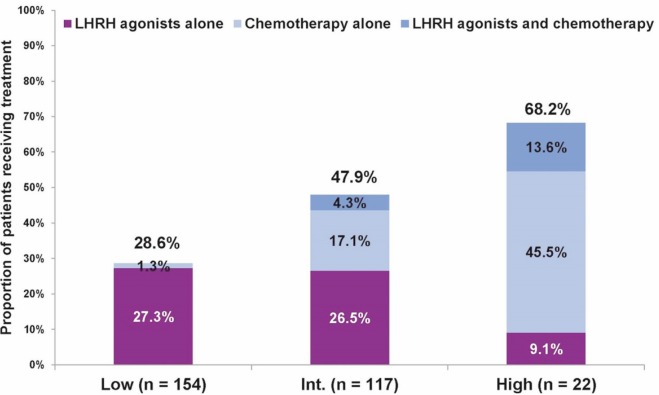
Treatment with LHRH agonists, chemotherapy, or both, in addition to tamoxifen by Recurrence Score category in patients ≤ 50 years. For treatment with LHRH agonists and/or chemotherapy: P < 0.0001 for comparing all three groups (chi-squared test).

Next, we evaluated the treatment approach in 293 patients aged ≤ 50 years, and assessed physicians’ choice of chemotherapy and/or LHRH agonists in addition to hormonal therapy with tamoxifen (LHRH agonists are considered as a potential substitute for chemotherapy in ER+ premenopausal women [12–17]) versus none. In this age group, overall, a more aggressive treatment approach was noted, and even in the low Recurrence Score group, close to one-third of patients received chemotherapy and/or LHRH agonists. Still, the proportion of patients receiving LHRH agonists and/or chemotherapy varied significantly across Recurrence Score groups (*P *< 0.0001 for comparison across the three groups; chi-squared test; Figure 2).

### Clinical outcomes and subsequent events

Median follow-up for the entire cohort was 26 months. We explored the rate of systemic recurrence by examining the MHS database to identify patients whose chemotherapy hospital services were provided more than one year from the date of Onco*type *DX testing. Six such patients (0.8%) were identified. These patients included one with ovarian cancer, one with colorectal cancer, one with acute myeloid leukaemia, and one with clear cell carcinoma of the uterus. In addition, two patients received late chemotherapy for locoregional recurrences in ipsilateral axillary lymph nodes. Notably, these two patients did not receive chemotherapy; one did not meet the MHS criteria due to involved axillary nodes and had a low Recurrence Score result, and the other was 43 years old with borderline eligibility (Grade 3, 1 cm) and an intermediate Recurrence Score result.

We also examined the MHS database to identify patients that had a mastectomy after more than six months from the date of Onco*type *DX testing. Five such patients (0.7%) were identified, of whom only only patient with high Recurrence Score result, who was treated with lumpectomy, radiation therapy, and chemotherapy, followed by tamoxifen, developed local breast recurrence 27 months after her primary surgery. Another patient, with intermediate Recurrence Score result who had lumpectomy followed by radiation, underwent contralateral mastectomy for newly diagnosed contralateral breast cancer 17 months from her initial surgery. The remaining three identified patients had mastectomies as a prophylactic measure or to facilitate obtaining symmetry in breast reconstruction.

Four patients died during follow-up: one due to advanced biliary tract malignancy, one from intracerebral haemorrhage, and one at the age of 86 with severe aortic stenosis; all were breast cancer free. The fourth patient who had an intermediate Recurrence Score result expired due to recurrent metastatic cervical cancer with extensive abdominal and pelvic involvement proved histologically at the time of small bowel resection surgery; several weeks prior to her death, the patient also had a biopsy-proven axillary nodal recurrence of ILC.

### Discussion

The current retrospective analysis of a prospectively defined cohort evaluated the association of Recurrence Score results, clinicopathological features, treatments received, and clinical outcomes in a large cohort of N0/Nmic ER+ primary breast cancer patients who underwent Onco*type *DX testing. The cohort included patients with low and intermediate risk pathological categories reflecting the MHS policy. As may be expected, the rate of high Recurrence Score results in our cohort (8%) was substantially lower than that observed in cohorts of ER+ node negative and node positive patients with unspecified histology grade (25–33%) [1, 5, 9]; still, high and intermediate Recurrence Score categories occurred in almost half of our population (46%).

We observed the associations between Recurrence Score results and age, tumour grade/size, and histology subtype. Specifically, high Recurrence Score was twice as common in younger compared with older age groups, consistent with the findings in [5]. Up to one-third of patients with Grade 3 ≤ 1 cm tumours had high Recurrence Score and a weak but statistically significant association between Recurrence Score and Ki-67 was observed, consistent with prior reports [18, 19]. Looking into the associations between histological subtypes and Recurrence Score yielded two observations: Recurrence Score distribution in patients with IDC with favourable features (mucinous, tubular, and medullary [11]) and IDC not specified was not statistically different, although the number of patients in the IDC favourable group was limited; and high Recurrence Score was extremely rare (2%) in patients with ILC histology, as compared with a rate of 8% in the IDC not specified group. The latter findings are supported by two other reports, the first by Asad *et al * [20] who observed a significant difference in Recurrence Score distribution between only eight patients with ILC and 77 patients with IDC with a higher proportion of low Recurrence Score patients in the ILC group; and the other by Baehner *et al * [21] who evaluated Recurrence Score results from more than 25,000 Genomic Health-tested commercial samples (and potentially associated with selection bias) and found a significantly lower median Recurrence Score result in ILC than IDC tumours (17.5 versus 18.2; *P* < 0.05).

This analysis is retrospective and conclusions regarding clinical outcomes and subsequent events are currently limited by a relatively short follow-up duration (median of 26 months). Nonetheless, the analysis has several strengths: data on treatments received and outcome were complete using MHS resources and all pathological reports were reviewed by an oncology physician. Furthermore, treatment decisions reflect national treatment trends as the majority of MHS patients are still treated in government hospitals due to a shortage of MHS oncologists (as no national treatment recommendations exist, all oncologists, regardless of HMO affiliation, are expected to use international treatment guidelines in their clinical practice). The logistic regression analysis demonstrated that both Recurrence Score category and age were independent factors impacting treatment decisions, with higher Recurrence Score and younger age increasing the likelihood of treatment with chemotherapy. Interestingly, our data suggest that in younger patients (≤ 50 years) with low Recurrence Score results, physicians may be inclined to administer more than ‘only tamoxifen’ and use an LHRH agonist despite the lack of evidence for its benefit [22]. In the high Recurrence Score group, approximately two-thirds of patients received chemotherapy, which is considerably less than optimal given the MHS eligibility criteria with request to discuss chemotherapy use in cases found to be at high recurrence risk. This apparent gap may suggest that, in some cases, the oncologist and/or patient may have changed their mind about chemotherapy treatment after the Recurrence Score results became available.

The rationale for the MHS policy is to identify patients whose lives may be saved by early chemotherapy use and who may not otherwise be considered for chemotherapy by the treating physicians. Indeed, follow-up data on this cohort, despite being limited (median of 26 months), with the various treatment approaches as detailed, showed only four locoregional recurrences, one in a patient borderline for eligibility and another in a noneligible patient. The MHS policy excludes patients with large Grade 3 tumours based on findings from the original Paik *et al *[1] report that assessed 668 tamoxifen-treated patients in the NSABP B-14 study and showed that high grade still had significant prognostic value in multivariable analysis, including the Recurrence Score. While in that report, concordance for histology grade was only 59–65% between two pathologists, it was highest for poorly differentiated tumours (kappa, 0.61) [1]. It should be noted, however, that low Recurrence Score may occur in about 20% of patients with Grade 3 tumours [1, 5], and that a Recurrence Score–pathology-clinical (RSPC) assessment was found to be more prognostic for distant recurrence than Recurrence Score alone [23]; nonetheless, the NSABP B20 data analysis suggests that Recurrence Score is still the best predictor of chemotherapy benefit (RSPC was not predictive of chemotherapy benefit [23]). We expect the data from a prospective trial that incorporates both Recurrence Score results and grade (the ongoing TAILORx trial), to best define their relative significance. Another molecular classification method using genomic grading index also supports the use of molecular classification mostly in Grade 2 tumours [24, 25]. To overcome potential inaccuracies in Grade 3 tumour readings, the MHS policy allows pathological reviews of all large Grade 3 tumours and approves Onco*type *DX testing if one of the reviewers considers the tumour to be of lower grade.

MHS, as a large HMO with limited resources, examines outcomes repeatedly, and reevaluates its policies accordingly. Currently, MHS is considering revising its Onco*type *DX eligibility criteria to exclude patients for whom testing is less likely to modify treatment decisions (i.e. patients with Grade 1 tumours and no micrometastases, were 0% are found to be high risk), in order to free resources that could be used to expand the eligibility criteria to potential other patient groups.

## Conclusions

Our results demonstrate that in a cohort of N0/Nmic ER+ primary breast cancer patients with low or intermediate risk by traditional parameters, 46% of patients had intermediate or high Recurrence Score results (38% and 8%, respectively). Recurrence Score findings along with patients’ age impacted treatment decisions, an approach that was potentially associated with favourable outcomes. Thus, using Onco*type *DX testing in this patient population may help to identify a clinically relevant proportion of patients that are likely to benefit from chemotherapy treatment.

## Conflicts of Interest

The authors have no conflicts of interest to declare.

## Figures and Tables

**Table 1.** Distribution of Recurrence Score categories by patient/tumour characteristics.

Parameter	Recurrence Score		
	Low	Intermediate	High
Age (*N* = 751)^a^			
18–44 years (*n* = 138)	62 (44.9%)	58 (42.0%)	18 (13.1%)
45–64 years (*n* = 485)	267 (55.1%)	184 (37.9%)	34 (7.0%)
≥ 65 years (*n* = 128)	78 (60.9%)	43 (33.6%)	7 (5.5%)
Histology subtype (*N* = 713)^b^			
IDC not specified (*n* = 590)	314 (53.2%)	228 (38.6%)	48 (8.1%)
IDC favourable (*n* = 21)	12 (57.1%)	7 (33.3%)	2 (9.5%)
ILC/mixed (*n* = 102)	67 (65.7%)	33 (32.4%)	2 (2.0%)
Grade/size combination (*N* = 605)^c^			
Grade 2, ≤ 1 cm (*n* = 113)	62 (54.9%)	47 (41.6%)	4 (3.5%)
Grade 3, ≤ 1 cm (*n* = 28)	7 (25.0%)	12 (42.9%)	9 (32.1%)
Grade 1, > 1 cm (*n* = 101)	60 (59.4%)	38 (37.6%)	3 (3.0%)
Grade 2, > 1 cm (*n* = 363)	200 (55.1%)	131 (36.1%)	32 (8.8%)
Ki-67 levels (*N* = 389)^d^			
≤ 15% (*n* = 251)	156 (62.2%)	86 (34.3%)	9 (3.6%)
> 15% (*n* = 138)	55 (39.9%)	59 (42.8%)	24 (17.4%)

a All patients. *P* = 0.002; Mantel-Haenszel Chi-squared test for association between age and Recurrence Score groups.

b MHS-eligible patients. *P *= 0.02 for overall differences between subtypes (Cochran-Mantel-Haenszel Row Mean Score test). Pairwise comparisons: *P *= 0.017 for IDC not specified v/s ILC/mixed (Cochran-Mantel-Haenszel Row Mean Score test using the Bonferroni correction). All other pairwise comparisons were nonsignificant.

c MHS-eligible patients with grade/size information. *P* < 0.0001 for overall differences between grade/size combinations (Cochran-Mantel-Haenszel Row Mean Score test). Pairwise comparisons: *P* < 0.0005 for Grade 3 and ≤ 1 cm v/s all other grade/size combinations (Cochran-Mantel-Haenszel Row Mean Score test using the Bonferroni correction). All other pairwise comparisons were nonsignificant.

d MHS-eligible patients with Ki-67 information. *P* < 0.0001 for overall differences between patients with Ki-67 ≤ 15% and > 15% (Cochran-Mantel-Haenszel Row Mean Score test).

**Table 2.** Odds ratios for receiving chemotherapy (logistic regression analysis) (*N* = 751).

Effect^a^	Odds Ratio	95% Wald confidence limits	*P*-value
*Recurrence Score category*			
Intermediate v/s low	11.0	4.2–28.4	< 0.0001
High v/s low	113.3	40.1–320.6	< 0.0001
High v/s intermediate	10.3	5.4–19.9	< 0.0001
*Age*			
45-64 years v/s 18-44 years	0.40	0.22–0.75	0.0043
≥ 65 years v/s 18-44 years	0.12	0.04–0.40	0.0006
≥ 65 years v/s 45-64 years	0.304	0.10–0.94	0.039

a Interactions between Recurrence Score categories and age groups were found to be nonsignificant
